# Comparison of efficacy and safety between native tissue repair and transvaginal mesh for uterine prolapse: a retrospective study

**DOI:** 10.3389/fsurg.2026.1840410

**Published:** 2026-06-16

**Authors:** Jing He, Huijuan Wang, Wei Wang, Na Dong

**Affiliations:** 1 Department of Gynecology III, Shijiazhuang Maternal and Child Health Hospital, Shijiazhuang, Hebei, China; 2 Operating Room, Shijiazhuang Maternal and Child Health Hospital, Shijiazhuang, Hebei, China; 3 The Sixth Department of Obstetrics, Shijiazhuang Maternal and Child Health Hospital, Shijiazhuang, Hebei, China

**Keywords:** complications, mesh, native tissue repair, recurrence, transvaginal surgery, uterine prolapse

## Abstract

**Background:**

The surgical treatment for patients with uterine prolapse usually adopts two methods: native tissue repair (NTR) and transvaginal mesh (TVM). However, there is still inconsistent conclusion regarding the efficacy and safety of these two methods at present.

**Objective:**

The aim is to compare the efficacy and safety of native tissue repair and transvaginal mesh in the treatment of symptomatic uterine prolapse.

**Methods:**

This study included 86 patients who were admitted to the hospital due to uterine prolapse from January 2022 to December 2024. According to the type of surgery they received, the patients were divided into two groups: the NTR group (*n* = 37) and the TVM group (*n* = 49). The perioperative indicators of the two groups were compared. The subjective symptom improvement was evaluated using the PFDI-20 score, the quality of life changes were reflected by the PFIQ-7 score, patient satisfaction was assessed using the PGI-I score, and the incidence of postoperative complications and the recurrence rate within one year were also compared.

**Results:**

The average operation time of the NTR group was 177.3 ± 30.51 min, while the operation time of the TVM group was shorter, at 148.8 ± 21.12 min (*P* < 0.001). In terms of hospital stay, the TVM group had a longer stay (6.49 ± 1.37 days), while the NTR group had a shorter stay (5.51 ± 1.22 days), and the difference was statistically significant (*P* < 0.001). The postoperative PFDI-20 and PFIQ-7 scores of the two groups improved significantly (*P* < 0.001), with the TVM group having a lower PFIQ-7 score (26.53 ± 6.37 vs. 30.84 ± 8.59, *P* = 0.009). The subjective satisfaction of the patients in both groups was similar, at 91.89% and 91.84% respectively, with no significant difference (*P* > 0.05). The overall complication rate of the TVM group was higher (30.61% vs. 10.81%, *P* = 0.028), but the recurrence rate was lower (4.08% vs. 18.92%, *P* = 0.029). Subgroup analysis of the TVM group showed that synthetic mesh was associated with a higher complication rate (34.15%) but lower 1-year recurrence (2.44%), whereas bioabsorbable mesh had a lower complication rate (12.50%) but higher recurrence (12.50%).

**Conclusion:**

Whether it is native tissue repair or vaginal mesh, both can improve the symptoms and overall health conditions of women with uterine prolapse. Vaginal mesh has a significant advantage in reducing the recurrence rate, but it also comes with a higher risk of mesh-related complications. Native tissue repair has advantages in terms of safety and complication rate, but its recurrence risk is relatively higher. Therefore, when choosing a specific surgical plan, it is necessary to comprehensively assess the individual circumstances of each patient and weigh the surgical effect against safety.

## Introduction

Uterine prolapse is an important subtype of pelvic organ prolapse, and its occurrence can significantly affect the quality of life and health status of women (1, 2). According to the FIGO (International Federation of Gynecology and Obstetrics) classification system for uterine prolapse, the severity of this condition is divided into different grades ranging from mild to severe. Among them, grade I represents mild prolapse, while grades III and above are classified as severe prolapse. Patients with different degrees of uterine prolapse face a series of problems, from mild discomfort to severe urination, defecation dysfunction and sexual dysfunction (3–5). As populations age and women focus more on their health, uterine prolapse is becoming more common, leading to a greater need for treatment (6, 7).

Surgical treatment is the main intervention for symptomatic moderate to severe uterine prolapse. Traditional native tissue repair relies on the patient's own fascia, ligaments and other tissue structures for anatomical reduction, which has the advantage of avoiding complications related to allogeneic material implantation, good long-term safety record, and relatively economic (8–10). However, some studies have pointed out that its postoperative recurrence rate is relatively high, especially in patients with severe prolapse or combined with multiple pelvic floor defects. It may be difficult to achieve lasting and stable anatomical support by autologous repair alone (11–13). In recent years, thanks to the increasing maturity of minimally invasive surgical techniques and the continuous progress of biomaterials, transvaginal mesh has become an important option for the treatment of uterine prolapse. By implanting synthetic or absorbable mesh materials into the pelvic floor tissue, vaginal mesh can provide more stable support for the uterus, help strengthen the pelvic floor structure, and effectively reduce the risk of recurrence after surgery (14, 15). Some early studies have shown that TVM has obvious advantages in improving the symptoms and quality of life of patients with pelvic organ prolapse, especially in the treatment of severe uterine prolapse and recurrent uterine prolapse (16, 17). However, mesh implantation may cause a series of complications, such as infection, mesh erosion, chronic pain, etc. Although the incidence of these complications is relatively low, once they occur, they will bring great pain to the patient, and even require a second operation to deal with them (18). At present, there are still some differences and controversies about the efficacy and safety of autologous tissue repair and mesh augmentation in the treatment of uterine prolapse. Therefore, the aim of this study is to systematically compare the efficacy and safety of autologous tissue repair and mesh augmentation in patients with uterine prolapse through a retrospective cohort analysis, in order to provide evidence-based evidence based on real-world clinical practice and provide reference for individualized treatment selection and clinical decision-making.

## Materials and methods

### Study design

From January 2022 to December 2024, this study retrospectively included 86 cases of uterine prolapse patients admitted to our hospital, forming a single-center cohort for analysis. All patients were diagnosed according to the clinical criteria for uterine prolapse, and a detailed assessment of the pelvic floor function was completed before the operation. Based on the different surgical methods, the patients were divided into two groups: the group with native tissue repair (37 cases) and the group with transvaginal mesh (49 cases). The choice of surgical method was not randomized but was a decision made by the physician and patient on the basis of shared decision making after thorough consultation of the potential benefits and risks of the two methods. Factors considered included the patient's age, general health, POP-Q stage, previous pelvic surgery history, personal preference, and surgeon expertise. The study protocol was approved by the institutional review board of our hospital. Given the retrospective nature of the study and the anonymization of all data, permission to wastage informed consent was obtained in accordance with the ethical principles of the Declaration of Helsinki.

### Regulatory context for transvaginal mesh in China

To be clear, the 2019 Food and Drug Administration (FDA) order to stop marketing of transvaginal mesh for pelvic organ prolapse applies only in the U.S. In China, the National Medical Products Administration has not issued a similar ban. With appropriate patient selection, adequate informed consent, and performed by a qualified surgeon, some types of transvaginal mesh, particularly type I macroporous polypropylene mesh, may still be legally used to treat moderate-to-severe or recurrent POP. All mesh products used in this study held valid NMPA registration certificates, and each patient was informed and aware of the mesh related risks.

### Inclusion and exclusion criteria

The study included patients who were 18 years of age or older; Uterine prolapse was diagnosed by clinical examination. Patients were treated with self-tissue repair or transvaginal mesh implantation. The clinical data were complete. Patients with concurrent genital-tract or other pelvic malignancies were excluded; Contraindications to surgery or anesthesia due to severe medical complications; Uncontrolled pelvic infection or systemic infection; Previous pelvic floor surgery or surgery related to uterine prolapse; Severe connective tissue disease, or long-term use of immunosuppressive agents; Patients with incomplete follow-up data or lost to follow-up.

### Operation method

Native tissue repair: Surgery was performed via the transvaginal approach. After routine preparation, a longitudinal incision was made in the anterior or posterior vaginal wall. The bladder and inferior rectal fascia were carefully dissected. For apical suspensions, high uterosacral ligament suspensions (HUSLS) were performed: bilateral uterosacral ligaments were identified at the level of the ischemia-spine and the vaginal apex/cervix was suspended with a long-term absorbable monofilament suture of polydioxanone (PDS II, 2-0, Ethicon, USA). Subsequently, the pubocervical fascia and rectovaginal fascia were folded using the same PDS II 2-0 suture with interrupted stitches for anterior or posterior vaginal closure. Cystoscopy was performed at the end of the procedure to confirm patency of the ureter. Transvaginal mesh: Surgery was also performed via the transvaginal route. The surgical procedure is similar to that of autologous tissue repair, but after the bladder and rectal space are separated, a pretailored mesh material is placed in the pelvic floor tissue to cover the anterior and posterior vaginal walls and the area around the cervix. The mesh is fixed with the surrounding fascia, muscles, and other tissues by sutures to provide additional support. Based on the patient's condition and the attending surgeon's experience, either a synthetic permanent mesh or a bioabsorbable mesh was selected. The synthetic mesh used was type I macroporous polypropylene monofilament mesh (Gynecare Gynemesh™, Ethicon, USA; or Ajust™, Bard, USA); the bioabsorbable mesh was a poly (lactic-co-glycolic acid) (PLGA) mesh (Gynemesh® Absorbable, Ethicon, USA). Aseptic principles were strictly followed during the operation to reduce the risk of infection.

### Data collection

The baseline data were extracted from the hospital's electronic medical record system and included information such as age, body mass index (BMI), menopause age, pregnancy history, number of deliveries, and comorbidities. The relevant information that needs to be collected during the perioperative period mainly includes: the duration of the surgery, the amount of blood loss during the operation, the duration of indwelling urinary catheter, the residual urine volume in the bladder after the operation, and the number of days spent in the hospital after the operation. Postoperative follow-up data included PFDI-20 subjective symptom score (19), PFIQ-7 quality of life score (20), PGI-I Patient Global impression of improvement score (21) and long-term complications. The higher the PFDI-20 score is, the more severe the pelvic floor dysfunction symptoms are; the higher the PFIQ-7 score is, the more significant the impact of the symptoms on the patient's quality of life becomes. PGI-I included 7 grades: very good, significantly better, slightly better, no change, slightly worse, much worse, and very bad. “Very good” or “significantly better” was defined as subjective satisfaction. All data were collected and collated by two independent researchers to ensure the accuracy and completeness of the data.

### Statistic analysis

GraphPad Prism 8.0 software was used for statistical analysis and data mapping. Measurement data were expressed as mean ± standard deviation. Independent sample *t* test was used for comparison between groups, and paired sample *t* test was used for comparison within groups. For count data, the *χ*^2^ test or Fisher's exact test was used for comparison between groups. Recurrence-free survival rates at 1 year were plotted with the use of Kaplan–Meier. For subgroup analyses comparing synthetic vs. bioabsorbable mesh within the TVM group, descriptive statistics were used given the small sample size in the bioabsorbable subgroup. *P* < 0.05 was considered statistically significant.

## Results

### Baseline characteristics

A total of 86 patients with uterine prolapse were included in the study, including 37 patients in the NTR group, 81.08% in POP-Q stage III and 18.92% in stage IV, with an average age of (60.86 ± 7.98) years. There were 49 patients in the TVM group, including 77.55% in stage III and 22.45% in stage IV, with an average age of (61.18 ± 7.47) years. Within the TVM group, 41 patients (83.67%) received synthetic permanent mesh and 8 patients (16.33%) received bioabsorbable mesh. There were no significant differences between the two groups in terms of age at baseline, body mass index (BMI), menopause age, number of deliveries, severity of prolapse, and major comorbidities such as hypertension and diabetes (*P* > 0.05), indicating that the two groups were comparable (Table 1).

**Table 1:** Comparison of general information of the two groups of patients.

Classification	NTR group (*n* = 37)	TVM group (*n* = 49)	*t*/*χ*^2^	*P* value
Age, years	60.86 ± 7.98	61.18 ± 7.47	0.190	0.850
BMI, kg/m^2^	23.80 ± 2.13	23.39 ± 2.21	0.866	0.389
Age of menopause, years	48.97 ± 3.29	49.65 ± 3.08	0.985	0.327
Gravidity, time	3.81 ± 0.78	3.78 ± 0.74	0.214	0.831
Parity, time	2.27 ± 0.69	2.18 ± 0.75	0.545	0.587
POP-Q staging, *n* (%)			0.159	0.690
III	30 (81.08)	38 (77.55)		
IV	7 (18.92)	11 (22.45)		
Hypertension, *n* (%)	18 (48.65)	26 (53.06)	0.164	0.685
Diabetes, *n* (%)	10 (27.03)	12 (24.49)	0.071	0.790

NTR, native tissue repair; TVM, transvaginal mesh; BMI, body mass index.

### Perioperative indicators

There were no significant differences between the two groups in terms of intraoperative blood loss (90.62 ± 13.75 milliliters vs. 87.98 ± 11.10 milliliters, *P* = 0.327), indwelling catheter duration (4.81 ± 1.27 days vs. 5.08 ± 1.15 days, *P* = 0.304), and postoperative residual urine volume (16.54 ± 6.56 milliliters vs. 15.29 ± 6.84 milliliters, *P* = 0.394). On the contrary, the operation time of the TVM group was significantly shorter than that of the NTR group (148.8 ± 21.12 min vs. 177.3 ± 30.51 min, *P* < 0.001), but the hospital stay was significantly longer (6.49 ± 1.37 days vs. 5.51 ± 1.22 days, *P* < 0.001) (Table 2).

**Table 2:** Comparison of perioperative indicators between the two patient groups.

Index	NTR group (*n* = 37)	TVM group (*n* = 49)	*t*	*P* value
Surgery time (min)	177.3 ± 30.51	148.8 ± 21.12	5.115	<0.001
Intraoperative blood loss (mL)	90.62 ± 13.75	87.98 ± 11.10	0.986	0.327
Duration of indwelling catheter (d)	4.81 ± 1.27	5.08 ± 1.15	1.035	0.304
Postoperative residual urine volume (mL)	16.54 ± 6.56	15.29 ± 6.84	0.857	0.394
Length of hospital stay (d)	5.51 ± 1.22	6.49 ± 1.37	3.43	<0.001

NTR, native tissue repair; TVM, transvaginal mesh.

### Postoperative quality of life

**Figure 1:**
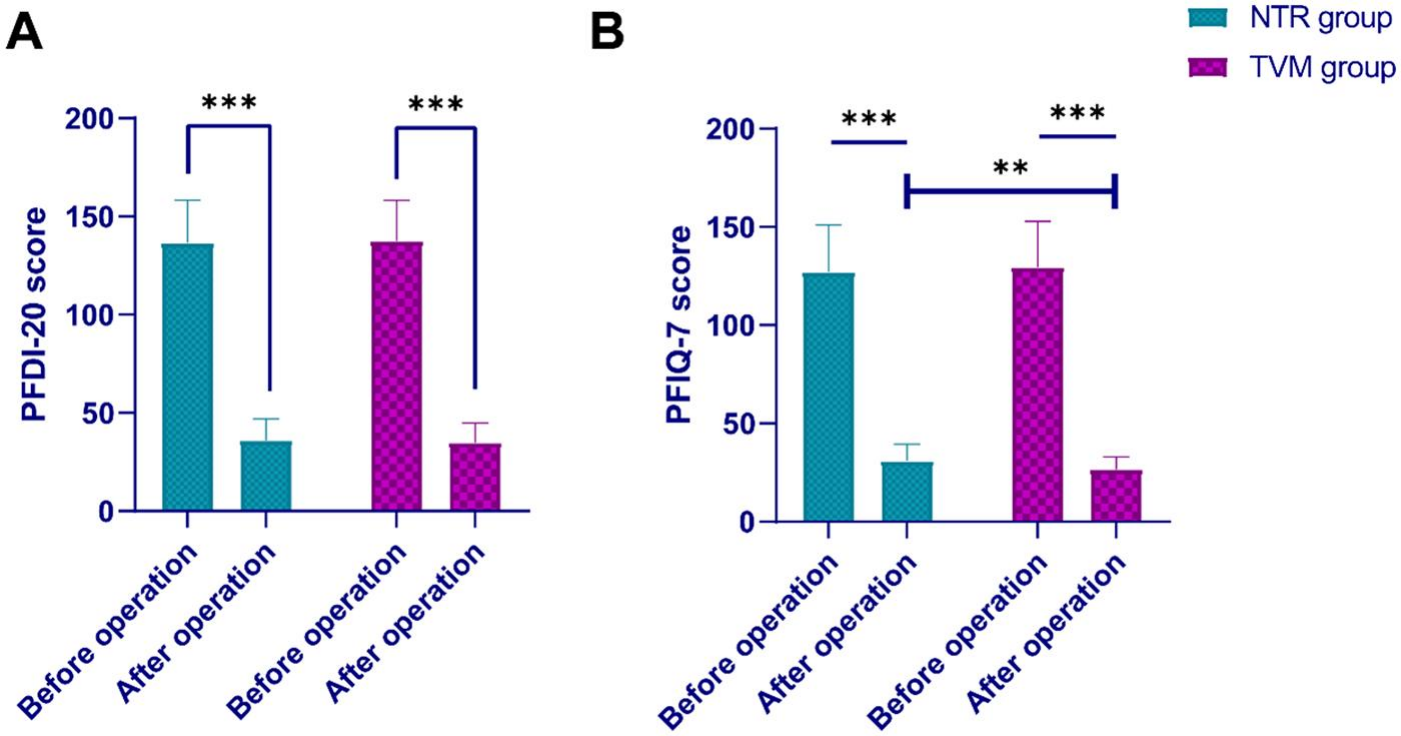
Comparison of PFDI-20 and PFIQ-7 scores between the two groups before and after operation. **(A)** PFDI-20 scores; **(B)** PFIQ-7 scores; NTR, native tissue repair; TVM, transvaginal mesh; PFDI-20, Pelvic Floor Distress Inventory-shortform20; PFIQ-7, Pelvic Floor Impact Questionnaire-short form 7.

Preoperative assessments indicated that both the NTR and TVM groups started with similar symptom and impact scores, as measured by the PFDI-20 (136.57 ± 36.11 vs. 137.65 ± 20.43, *P* = 0.813) and PFIQ-7 (127.14 ± 23.99 vs. 129.55 ± 23.29, *P* = 0.639). The postoperative scores of the two groups were significantly improved when compared with those before operation (*P* < 0.001). The postoperative PFDI-20 and PFIQ-7 scores of NTR group were 36.11 ± 10.81 and 30.84 ± 8.59, respectively. In TVM group, the above two scores were 34.84 ± 10.14 and 26.53 ± 6.37, respectively. Although the difference in postoperative PFDI-20 scores between the groups was not significant (*P* = 0.577), the TVM group reported a noticeably lower PFIQ-7 score compared to the NTR group (*P* = 0.009) (Figure 1).

### Subjective satisfaction rate

**Figure 2:**
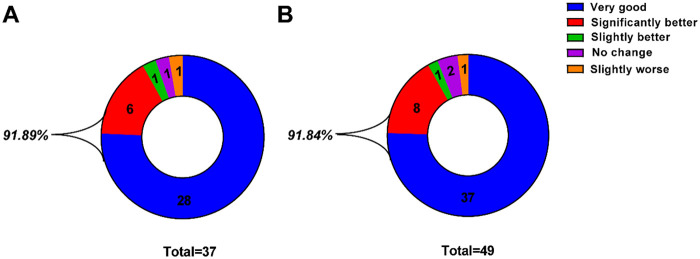
Subjective satisfaction rate of two groups. **(A)** NTR group; **(B)** TVM group; NTR, native tissue repair; TVM, transvaginal mesh.

At 12-month follow-up, patients in both groups reported the following results: In NTR group, 28 patients felt good recovery, 6 patients felt significant improvement, 1 patient felt slightly better, 1 patient felt no change, and 1 patient felt slightly worse. The other patients did not have other conditions, and the subjective satisfaction was 91.89% (34 of 37 cases). In the TVM group, 37 patients felt good, 8 reported significant improvement, 1 reported slight improvement, 2 reported no change, and 1 reported slight deterioration. No other reactions were recorded. The subjective satisfaction rate was 91.84% (45 out of 49). There was no significant difference in satisfaction between the two groups (*P* > 0.05) (Figure 2).

### Postoperative complications

During the 1-year follow-up period, the overall complication rate in the transvaginal mesh group (TVM) was 30.61%, significantly higher than that in the native tissue repair group (NTR) at 10.81% (*P* = 0.028). The main complications in the mesh group included mesh exposure/erosion (6.12%), chronic pain (6.12%), and infection (6.12%), while the main complication in the native tissue repair group was pelvic hematoma (5.40%). It is noteworthy that mesh exposure/erosion and chronic pain are specific complications of TVM. When complications were stratified according to the Clavien-Dindo classification, most were mild to moderate. In TVM group, there were 7 cases of grade I complications, 7 cases of grade II complications, and 1 case of grade III complications. Therefore, the incidence of serious complications requiring surgical treatment in the TVM group was 2.04% (1/49). In NTR group, all 4 cases had complications of grade I and did not need reoperation (Table 3). When the TVM group was stratified according to the type of mesh, the total complication rate was 34.15%(14/41) in the synthetic mesh group and 12.50% (1/8) in the bioresorbable mesh group. Only the synthetic mesh group had mesh exposure/erosion (3/41,7.32%) (Table 4).

**Table 3:** Comparison of postoperative complications between the two groups.

Classification	NTR group (*n* = 37)	TVM group (*n* = 49)	Clavien-Dindo grade	*χ* ^2^	*P* value
New onset SUI	1 (2.70)	2 (4.08)	I		
New onset UUI	0 (0.00)	1 (2.04)	II		
New onset voiding dysfunction	0 (0.00)	1 (2.04)	I		
New onset constipation	0 (0.00)	1 (2.04)	II		
Chronic pain	/	3 (6.12)	II (2), I (1)		
Mesh exposure/erosion	/	3 (6.12)	III (1), I (2)		
Exposure of sutures	1 (2.70)	0 (0.00)	I		
Pelvic floor hematoma	2 (5.40)	1 (2.04)	I		
Infection	0 (0.00)	3 (6.12)	II		
Overall	4 (10.81)	15 (30.61)		4.802	0.028

NTR, native tissue repair; TVM, transvaginal mesh; SUI, stress urinary incontinence; UUI, urge urinary incontinence.

**Table 4:** Subgroup analysis of transvaginal mesh patients by mesh type.

Mesh type	Synthetic permanent	Synthetic permanent	Bioabsorbable
Brand (manufacturer)	Gynecare Gynemesh™ (Ethicon)	Ajust™ (Bard)	Gynemesh® Absorbable (Ethicon)
Material	Polypropylene	Polypropylene	PLGA
Filament type	Monofilament	Monofilament	Multifilament
Pore size	Macroporous (>75 *μ*m)	Macroporous (>75 μm)	Not applicable
Number of patients (%)	29 (59.18)	12 (24.49)	8 (16.33)
Overall complications (%)	10 (34.48)	4 (33.33)	1 (12.50)
Mesh exposure/erosion (%)	2 (6.90)	1 (8.33)	0 (0.00)
1-year recurrence (%)	1 (3.45)	0 (0.00)	1 (12.50)

### Recurrence rate

**Figure 3:**
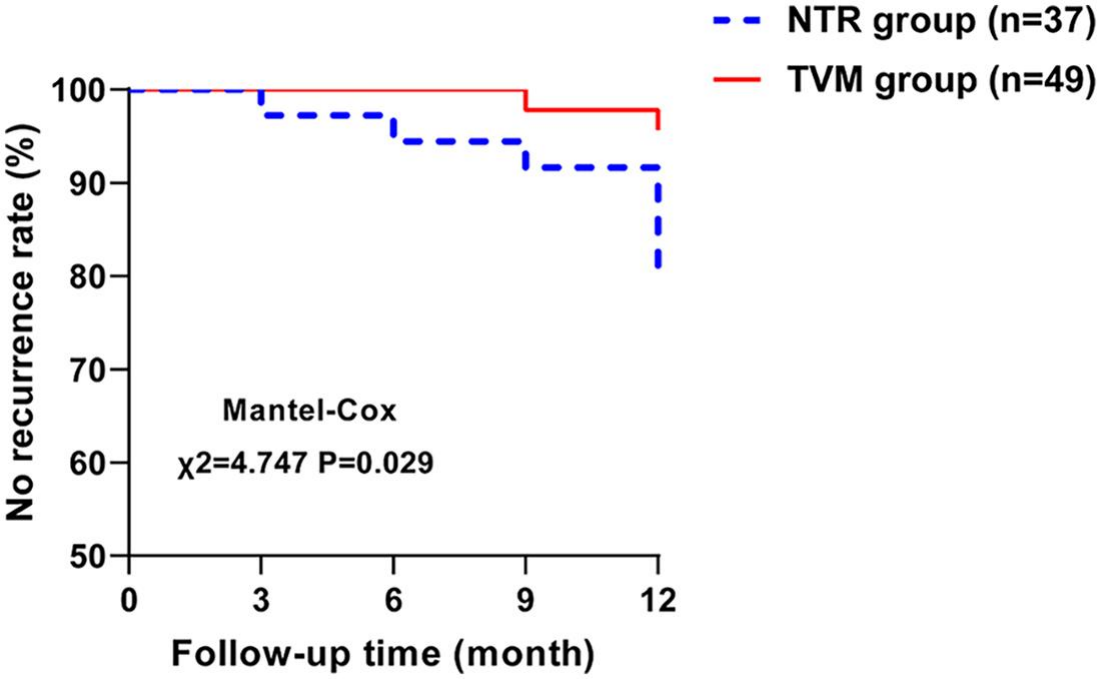
Kaplan–Meier curve of recurrence free rate at 1-year post operation in both groups. NTR, native tissue repair; TVM, transvaginal mesh.

Recurrence was defined as any descent of the leading edge beyond the hymen (Stage II or greater) or bothersome bulge symptoms. In this study, the recurrence rate in the TVM group was only 4.08% (2 out of 49 patients), while it was 18.92% in the NTR group (7 out of 37 patients). The Kaplan–Meier survival analysis revealed that the recurrence-free survival rate in the TVM group was significantly higher than that in the NTR group, and the difference was statistically significant (*χ*² = 4.747, *P* = 0.029) (Figure 3). In the TVM subgroup analysis, the 1-year recurrence rate was 2.44% (1/41) for synthetic mesh and 12.50% (1/8) for bioabsorbable mesh (Table 4).

## Discussion

Through a comprehensive comparison, this retrospective cohort study found that both native tissue repair and transvaginal mesh can effectively improve the pelvic floor symptoms and quality of life of patients in the treatment of uterine prolapse. However, there are significant differences between the two procedures in the duration of operation, length of hospital stay, incidence of complications, and postoperative recurrence rate. TVM group had shorter operation time, lower recurrence rate and better improvement of quality of life score, but higher incidence of mesh related complications and longer hospital stay. These findings provide an important basis for clinical selection of individualized surgical procedures.

The operation time of TVM group was significantly shorter than that of NTR group, which may be related to the standardized support structure provided by the mesh material, which reduced the complexity of intraoperative tissue suture and reinforcement. Of note, although the operative time was significantly shorter in the TVM group than in the NTR group, the postoperative hospital stay was significantly longer. This phenomenon is related to the more stringent monitoring of voiding function, the need for pain management after TVM, and the preventive observation of early complications of mesh. The length of surgery is not equivalent to the speed of postoperative recovery, especially in surgery involving implants, and the complexity of postoperative management and conservative strategies may have a greater impact on the length of hospital stay. Both surgeries significantly reduced the postoperative PFDI-20 and PFIQ-7 scores, which was in line with their therapeutic goals and confirmed their efficacy in alleviating symptoms and improving quality of life. The score of postoperative quality of life questionnaire (PFIQ-7) in TVM group was significantly lower than that in NTR group. This means that transvaginal mesh may be better than traditional surgery to alleviate the impact of pelvic floor dysfunction on patients' daily life. The additional support provided by the mesh may be more conducive to restoring the lasting stability of the pelvic floor structure, resulting in better functional experience, which is consistent with the conclusions of some studies (22, 23). It is worth noting, however, that the difference in PFIQ-7 scores between the two groups was 4.31, which was lower than the minimum clinically important difference (MCID) reported by PFIQ-7. Therefore, although TVM may have a statistical advantage in quality of life scores, its clinical advantage over autologous tissue repair remains uncertain. In terms of safety, this study found that the total incidence of complications in TVM group was significantly higher than that in NTR group at 1 year after operation. Complications specific to the TVM group included mesh exposure, erosion, chronic pain, and infection, which were all related to the implantation of allogeneic materials (24, 25). These complications may require secondary surgery to manage (26). In contrast, the NTR group demonstrated certain advantages in terms of safety, mainly manifested by a lower incidence of pelvic hematoma. This result indicates that when choosing a surgical treatment plan, both efficacy and safety should be taken into account comprehensively. It is worth noting that approximately one-third of women who undergo pelvic organ prolapse surgery will experience recurrence (27). In this study, the 1-year recurrence rate of the TVM group was significantly lower than that of the NTR group, and the Kaplan–Meier curve indicated that the recurrence-free survival rate of the TVM group was higher. This finding supports the advantage of TVM in providing durable support and reducing anatomical recurrence, especially in patients with severe prolapse (stage III-IV) or high risk for recurrence (28). However, it should be noted that the follow-up time of this study was only 1 year, and the long-term recurrence still needs to be further observed.

An important finding of the subgroup analysis was that mixing synthetic permanent mesh and bioresorbable mesh in the TVM group would mask important differences. Overall complication rates were higher with synthetic mesh (34.15% vs. 12.50%), but 1-year recurrence rates were lower (2.44% vs. 12.50%), whereas the opposite was the case with bioresorbable mesh. This supports the view that the two grid types have markedly different risk-benefit profiles and should not be viewed as interchangeable. Mesh exposure/erosion events occurred only in the synthetic mesh subgroup (3/412,7.32%), which is consistent with the known long-term performance of permanent polypropylene mesh. Furthermore, the choice of suture material for the NTR group deserves careful discussion. PDS II (polydioxanone, 2-0, Ethicon), a long-term absorbable monofilament suture, was used in this study, which retained approximately 60 to 70% tensile strength at 6 weeks and was fully absorbed at 180 to 210 days. This provides adequate support during the critical early healing phase (6–8 weeks). The 18.92% recurrence rate could have been higher with a short-term absorbable suture such as Vicryl, which loses maximum strength at 3–4 weeks. Thus, the recurrence rate in the NTR group should not be interpreted as a failure of the autologous tissue repair approach *per se*, but rather reflect real-world results that can be achieved using good long-term absorbable sutures. Future studies should explicitly report the suture type to allow accurate comparisons between studies.

The results of this study corroborate the important FDA warning and the delisting of some mesh products, highlighting ongoing safety concerns (29). The choice of surgical methods should be individualized and weigh the efficacy and safety. In this cohort, TVM was associated with a lower 1-year recurrence rate and better improvement in PFIQ-7 scores, but a significantly higher overall complication rate. Therefore, TVM may be considered for certain patients with a high risk of recurrence after adequate preoperative consultation about the risks associated with the mesh. However, according to current international guidelines, TVM should not be advocated as a routine option for young patients based on quality of life expectations alone. Autologous tissue repair remains a safer alternative, especially for patients with a lower risk of recurrence or a higher concern about complications. The final decision must take into account a combination of patient preference, prolapse severity, comorbidities, and fully informed consent.

### Limitations

Although this study provides some evidence-based evidence on the efficacy and safety of NTR and TVM in the treatment of uterine prolapse, there are still some limitations. This study is a single-center retrospective analysis, which may have a certain degree of selection bias and information bias. Additionally, the follow-up period is relatively short, and the long-term efficacy and related complications could not be evaluated. The study did not perform propensity score matching to reduce selection bias, and thus could not rule out residual confounding caused by unmeasured factors such as the degree of lateral defect, the surgeon's case burden, or specific patient preferences. Moreover, subgroup analyses of synthetic and bioresorbable patches were limited by the small sample size (*n* = 8) in the bioresorbable patch group, which precluded robust statistical comparisons. These results should be considered exploratory. In the future, we will need to conduct more large scale, multi center and long term follow-up studies to gain a deeper understanding of the effects of different patch materials, surgical methods, and individual patient characteristics on the treatment outcomes.

## Conclusion

Both native tissue repair and transvaginal mesh are effective surgical methods for the treatment of moderate and severe uterine prolapse. TVM has certain advantages in reducing postoperative recurrence rate and improving the quality of life of patients, but it has a high incidence of complications. NTR has good safety, but the recurrence rate is relatively high. The specific choice of mesh and suture material significantly affected outcomes. In clinical practice, doctors need to carefully choose the appropriate surgical method according to the specific conditions of patients, comprehensive consideration of surgical efficacy and safety.

## Data Availability

The raw data supporting the conclusions of this article will be made available by the authors, without undue reservation.
